# Personalized Web-Based Advice in Combination With Well-Child Visits to Prevent Overweight in Young Children: Cluster Randomized Controlled Trial

**DOI:** 10.2196/jmir.7115

**Published:** 2017-07-27

**Authors:** Amy van Grieken, Eline Vlasblom, Lu Wang, Maaike Beltman, Magda M Boere-Boonekamp, Monique P L'Hoir, Hein Raat

**Affiliations:** ^1^ Department of Public Health Erasmus University Medical Center Rotterdam Netherlands; ^2^ TNO Child Health Leiden Netherlands; ^3^ IGS Institute for Innovation and Governance Studies Department Health Technology and Services Research University of Twente Enschede Netherlands; ^4^ Department of Agrotechnology and Food Sciences Subdivision Human Nutrition Wageningen University & Research Wageningen Netherlands

**Keywords:** healthy lifestyle, child, preschool, parenting, child health, intervention study, eHealth, randomized controlled trial, body mass index

## Abstract

**Background:**

Overweight is a major health issue, and parent-targeted interventions to promote healthy development in children are needed.

**Objective:**

The study aimed to evaluate E-health4Uth Healthy Toddler, an intervention that educates parents of children aged 18 to 24 months regarding health-related behaviors, as compared with usual care. The effect of this intervention on the following primary outcomes was evaluated when the children were 36 months of age: health-related behaviors (breakfast daily, activity and outside play, sweetened beverage consumption, television (TV) viewing and computer time), body mass index (BMI), and the prevalence of overweight and obesity.

**Methods:**

The BeeBOFT (acronym for breastfeeding, breakfast daily, outside playing, few sweet drinks, less TV viewing) study is a cluster randomized controlled trial involving 51 Youth Health Care (YHC) teams. In total, 1094 parents participated in the control group, and 1008 parents participated in the E-health4Uth Healthy Toddler intervention group. The intervention consisted of Web-based personalized advice given to parents who completed an eHealth module and discussion of the advice during a regular well-child visit. In this study the eHealth module was offered to parents before two regular well-child visits at 18 and 24 months of age. During the well-child visits, the parents’ personalized advice was combined with face-to-face counseling provided by the YHC professional. Parents in the control group received usual care, consisting of the regular well-child visits during which general information on child health-related behavior was provided to parents. Parents completed questionnaires regarding family characteristics and health-related behaviors when the child was 1 month (inclusion), 6 months, 14 months, and 36 months (follow-up) of age. The child’s height and weight were measured by trained health care professionals from birth through 36 months of age at fixed time points. Multilevel linear and logistic regression models were used to evaluate the primary outcomes at 36 months of age.

**Results:**

At 36 months, we observed no differences between health-related behaviors of children, BMI or the percentage of children having overweight or obesity in the control and intervention group (*P*>.05). An analysis of the intervention effect revealed that boys benefited from eating breakfast daily, non-Dutch children spent more time being active or playing outdoors, children of low-educated parents and of overweight and obese mothers spent less time watching TV or using the computer, and children of normal weight mothers drank less sweetened beverages (*P*<.05) compared with the control group.

**Conclusions:**

The E-health4Uth Healthy Toddler intervention resulted in small improvements in health-related behaviors among subgroups but had no significant effects with respect to the children’s BMI. We conclude that the E-health4Uth Healthy Toddler intervention may be useful for pediatric health care professionals in terms of providing parents with personalized information regarding their child’s health-related behaviors.

**Trial Registration:**

Netherlands Trial Register: NTR1831; http://www.trialregister.nl/trialreg/admin/rctview.asp?TC=1831 (Archived by WebCite at http://www.webcitation.org/6mm5YFOB0)

## Introduction

### Background

In 2009, 1.8% of the Dutch boys and 2.2% of the Dutch girls in the age group of 2 to 21 years were classified as being obese, and 12.8% of the boys and 14.8% of the girls were classified as being overweight [1]. Compared with 1980, this is a 4- to 6- fold increase in obesity prevalence and a 2- to 3- fold increase in overweight prevalence among Dutch youth [1]. However, some studies suggest that the prevalence may have stabilized in some parts of the Netherlands, similar to data obtained from the United States [2,3]. Nevertheless, it remains unclear precisely which interventions are responsible for this stabilization [2]. Therefore, evaluating intervention programs may reveal how the prevalence of overweight and/or obese children can be decreased [2].

In the Netherlands, Youth Health Care (YHC) is a free program that monitors each child’s health and development and helps families promote healthy behaviors and prevent disease. These benefits are offered to parents and their children in the form of appointments with YHC at set intervals beginning in the child’s first year of life; in total, parents are offered a maximum of 11 well-child visits per child. During a well-child visit, growth and development of the child is assessed and discussed using standardized measures and protocols. Although voluntary, approximately 95% of parents in the Netherlands participate in this program [4]. The YHC uses a consensus-based overweight detection and prevention protocol [5,6], which uses international age- and gender-specific body mass index (BMI) cutoff values to evaluate a child’s weight status [7]. The prevention protocol provides the YHC professional (eg, community physician or nurse) with the means to offer primary and secondary prevention strategies to parents; parents of overweight children can also be offered additional counseling [5]. These prevention strategies can be translated into interventions suitable for use by the YHC to help prevent overweight among children. One such intervention is the E-health4Uth Healthy Toddler intervention [8], which gives parents of young children a Web-based eHealth module providing personalized education regarding their child’s nutritional habits and physical activity. This eHealth module is combined with face-to-face counseling by the YHC professional to the parents during the regular well-child visits [9-11]. During a pilot study wherein E-health4Uth Healthy Toddler intervention was developed and evaluated, results indicated that parents generally appreciate the advice they received from the eHealth module. Therefore, YHC professionals indicated that an eHealth module in combination with counseling could be integrated into their daily practice [12].

The E-health4Uth Healthy Toddler intervention is based on the following theories of behavior change: social-ecological theories and models, including the theory of planned behavior [13], the social learning model [14], and the McGuire communication model [15]. Personalized tailored advice regarding the child’s health-related behaviors is generated based on the answers that parents provide when completing the assessment questionnaire of the eHealth module. The advice is based on most recent guidelines for child health-related behavior. In this study, the eHealth module was offered online to parents before two regular well-child visits at a preventive YHC organization at 18 and 24 months of age. During the well-child visit of approximately 20 minutes, the parents’ personalized tailored advice was combined with face-to-face counseling provided by the YHC professional (eg, community physician or nurse). In general, in order to change health behavior, the use of Web-based and tailored eHealth tools may enhance intervention effectiveness [11,16,17]. A review by Hammersley et al [18] suggested that parent-focused overweight and obesity eHealth interventions can result in improvements in dietary or physical activity outcomes.

### Objective of This Study

The aim of this study was to compare the effects of applying the E-health4Uth Healthy Toddler intervention versus usual care (control) by assessing the following primary outcomes: breakfast daily, activity and outdoor play, sweetened beverages, screen time (ie, television (TV) watching and/or computer use), BMI, and the prevalence of overweight/obesity [8]. Specifically, we tested the following two hypotheses: (1) that children in the E-health4Uth Healthy Toddler intervention group eat breakfast daily, are more active, consume fewer sweetened beverages, and spend less time in front of the TV and/or computer at follow-up (at 36 months of age); and (2) that children in the intervention group would have lower BMI and a lower prevalence of overweight and/or obesity at follow-up. We also examined the effects of the following possible moderating factors: the child’s gender, the child’s ethnic background, maternal education level, and maternal weight status.

## Methods

### Study Design

In 2009, 50 preventive YHC organizations in the Netherlands were invited to participate in a 3-armed cluster randomized controlled trial (RCT) entitled the BeeBOFT (acronym for breastfeeding, breakfast daily, outside playing, few sweet drinks, less TV viewing) study (Netherlands Trial Register: NTR1831) [8]. A total of 10 organizations participated, including a total of 51 YHC teams. Each YHC organization serves a region of the Netherlands, and each team within an organization serves one or more municipalities of the region [4]. A team is comprised of a YHC physician, nurse, and assistant.

Within each of these ten organizations, the teams were randomly assigned to one of the following three groups using a computerized random allocation generator: the control group (n=17 teams), the E-health4Uth Healthy Toddler intervention group (n=17 teams), and the BeeBOFT + intervention group (n=17 teams). The E-health4Uth Healthy Toddler intervention group invited parents, at the child age of 18 and 24 months, to complete a Web-based eHealth module providing tailored health education regarding healthy child nutrition and activity behaviors and to discuss this advice during the regular well-child visit with a YHC professional. Therefore, Internet literacy was an implicit eligibility criterion. The YHC teams allocated to the BeeBOFT + intervention group focused on effective child rearing by parents from birth onwards by enlarging parental skills concerning healthy behavioral lifestyle habits. Parents in the control group received usual care, consisting of the regular well-child visits during which general information is provided with regard to health and development of the child. In this study, we focus only on the effects of the E-health4Uth Healthy Toddler intervention compared with usual care.

It is important to note that the YHC professionals and parents were not blinded with respect to the groups. The research proposal was reviewed by the Medical Ethics Committee of the Erasmus University Medical Center. On the basis of their review, the Committee concluded that the Dutch Medical Research Involving Human Subjects Act (in Dutch: Wet medisch-wetenschappelijk onderzoek met mensen) did not apply to this research proposal. The Medical Ethics Committee therefore had no objection to the execution of this study (proposal number MEC-2008-250). Further details regarding the study design and interventions have been published previously [8].

### Procedure and Study Population

From January 2009 through September 2010, the 51 participating YHC teams invited the parents of 7985 children to participate in the BeeBOFT study during their first YHC well-child visit, which was conducted at the parents’ home approximately 2-4 weeks after the birth of the child. Parents were requested to provide written informed consent to participate in the 3-year study. In total, 3003 parents agreed to participate in the BeeBOFT study and provided written informed consent (a participation rate of 37.61%; 3003/7985). At inclusion, a questionnaire was completed by the parents; 3 participants did not complete this questionnaire. This questionnaire contained items regarding the pregnancy (eg, duration and complications), childbirth (eg, complications and height and weight at birth), and family demographics (eg, the country of birth of both parents and grandparents, parents’ education level, and number of siblings).

When the child was 6, 14, and 36 months of age, all parents participating in the BeeBOFT study were invited to complete a more extensive questionnaire containing items regarding the child’s health-related behaviors, determinants of these behaviors, and the child’s health-related quality of life (HRQoL). The questionnaires could be completed on paper or online by either the mother or the father. The response rates at the three ages were 77.62% (2331/3003), 77.20% (2318/3003), and 73.46% (2206/3003), respectively.

**Figure 1:**
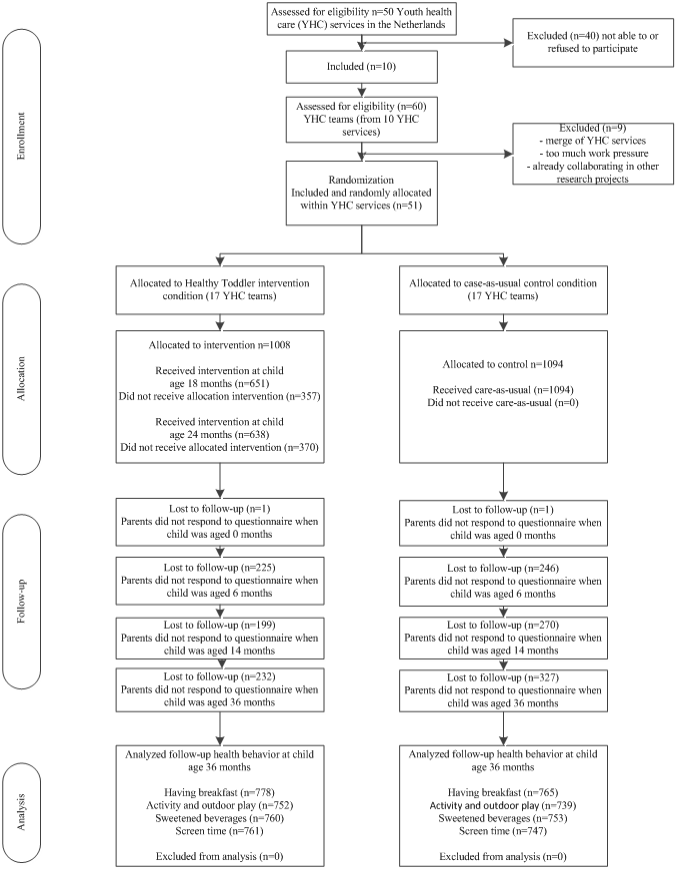
Flow of participants.

Here, we present our analysis of the effects comparing children in the E-health4Uth Healthy Toddler intervention group (n=1008 parents) and the control group (n=1094 parents) on the primary outcomes measured when the children were 36 months of age. The results obtained comparing children in the BeeBOFT + intervention group (n=991 parents) and the control group will be reported elsewhere. An overview of the YHC teams and the study participants is presented in Figure 1.

### The E-health4Uth Healthy Toddler Intervention

The E-health4Uth Healthy Toddler intervention has been described in detail by Raat et al [8]. Screenshots of the intervention are available in the Multimedia Appendices 1 and 2. In short, the E-health4Uth Healthy Toddler intervention provides parents with customized advice regarding key health behaviors designed to prevent childhood overweight. The following four key behaviors are targeted by the intervention: (1) the promotion of eating breakfast daily, (2) the stimulation of daily exercise and outdoor playing, (3) discouraging the consumption of sweetened beverages, and (4) discouraging TV viewing and computer use. The intervention is based on theories of behavior change (eg, the theory of planned behavior [13] and the social cognitive theory [14]) and information processing theories (eg, the McGuire communication model [15]).

Parents allocated to the intervention group received an invitation to visit the E-health4Uth Healthy Toddler intervention website [19] and complete the eHealth module online one month before the regular well-child visits scheduled for when the child was approximately 18 and 24 months of age. Before the pre-24-month invitation was sent, the eHealth module was evaluated to ensure that it was suitable for the parents of 24-month-old children; no changes were necessary.

As a first step, parents completed the assessment questionnaire; the questions in this questionnaire were based on previous research [20]. The assessment questionnaire started with 4 general questions (ie, gender of the child, age, date of upcoming well-child visit, who completed the questionnaire) used to personalize the advice. Thereafter, the assessment questionnaire assessed child health-related lifestyle behavior. The number of questions parents received depended on the responses provided by the parents (eg, if the parent indicated that the child did not play outside, there would be no follow-up question). There were a maximum of 5 questions on TV viewing and computer gaming, 5 questions on physical activity, 4 questions on breakfast, 11 questions on sweetened beverages, 9 questions on snacking, and 2 questions on fruit. Seven questions assessed parental attitude towards the behavior (eg, do you think your child drinks too many sweetened beverages). The answers to the assessment questionnaire were used to generate the tailored advice. A total of 96 messages were developed for the tailored advice, which could be combined in various ways, based on the parents’ responses. If the health-related behaviors were consistent with established guidelines, the parents received positive feedback [5]. The tailored advice could be read directly online, and parents were able to print the advice.

After reading the tailored advice, parents could make an implementation-intention plan in which they could specify actions (eg, what, when, and where to improve child health-related behavior). The tailored advice and implementation plan was sent by email to the parents. During the subsequent well-child visit, the advice was discussed with the YHC professional. The YHC professional, with permission of the parents, also had access to the advice parents received. During these well-child visits at approximately 18 and 24 months of age, the YHC professionals prescribed intervention conditions based on motivational interviewing techniques to help parents change their child’s health-related behaviors [17,21]. At the start of the study, the YHC professionals assigned to perform the intervention received a half-day training session on motivational interviewing techniques (eg, creating awareness, stimulating goal setting). The YHC professionals worked in teams (eg, community physician, nurse), and each team within a preventive YHC organization served one or more municipalities of the region. Each well-child visit was scheduled to last approximately 20 minutes. One month after the well-child visit, the parents received a follow-up email with the advice attached for their convenience and to strengthen the message.

The intervention software (TailorBuiler) was developed by OverNite Software Europe (OSE, Geleen, the Netherlands).

### Control Group (Care-As-Usual)

In the control group, the parents received usual care, which included regular YHC well-child visits. The YHC professionals in the control group provided care in accordance with the YHC Overweight Prevention Protocol [5]; they did not receive the information or instructions that were provided to the E-health4Uth Healthy Toddler intervention group [8].

### Measures

#### Primary Outcome Measures

Health-related behaviors were assessed using the BeeBOFT study questionnaires. All questions were adapted from Dutch questionnaires that were used in previous studies [21-23]. The parents reported the following aspects of their child’s health-related behavior during the previous 4 weeks: (1) eating breakfast daily, (2) daily exercise and outdoor play, (3) drinking sweetened beverages, and (4) TV viewing and computer use. For details regarding the timing of these assessments and the questions used, see Multimedia Appendix 3.

Daily breakfast was assessed by asking how many days of the week the child ate breakfast. Parents were instructed to report how many days per week and how much time per day their child spent being active and playing outdoors. Activity and outdoor play were added up to calculate an average hours of activity per day. Parents were instructed to indicate the number of glasses of sweetened beverages their child drank per weekday and weekend day. Examples of sweetened beverages were provided. Daily consumption in glasses per day was calculated. Finally, TV viewing and computer use were assessed by asking parents to report the average number of hours their child spent watching TV and/or using the computer per weekday and weekend day. Screen time in hours per day was calculated by adding up TV viewing time and computer time. At each YHC well-child visit, the child’s height and weight were measured in accordance with standardized protocols [6]. In addition, any missing birth weight data were obtained from the parent-reported questionnaire. BMI was calculated by dividing the child’s weight (in kilograms) by the child’s height (in meters squared). Each child was classified as being “normal” weight, overweight, or obese in accordance with international age- and gender-specific cutoff values [7]. A dichotomous variable was created of “normal weight” versus “overweight and obese.” In addition, Dutch reference values for children’s height and weight measured in 1980 were used to calculate the BMI standard deviation score (BMI-SDS) [1].

#### Other Measures

The following measures were included to describe the study population, evaluate potential confounding factors [8], and to evaluate potential moderating factors [8]. Data regarding the child and parents’ sociodemographic characteristics were obtained from the inclusion questionnaire. Any missing data were obtained using data collected from the questionnaire when the child was 6 months of age. In the questionnaire, the respondents reported their country of birth, the other parent’s country of birth, and the country of birth of the child’s grandparents. The child’s ethnic background was defined as follows in accordance with the definition established by Statistics Netherlands: a parent was classified as non-Dutch if one of his/her own parents was born outside the Netherlands. If one or both of the child’s parents were classified as non-Dutch, that child’s ethnic background was non-Dutch [24]. The respondents also reported the education level of both parents; education level was classified as one of the following three categories: low (no education or primary school), middle (high school or secondary education level), or high (junior college or university education) [25]. The parents’ employment status (unemployed or employed) and height and weight were also assessed. The parents’ BMI was calculated by dividing the parent-reported weight (in kilograms) by the height (in meters squared). Each parent was classified as either “normal” weight (BMI <25.0) or overweight and obese (BMI ≥25.0). Respondent self-rated health was assessed with a single item (ie, In general, would you say your health is excellent, very good, good, fair, or poor? Response options: (1) Excellent, (2) Very good, (3) Good, (4) Fair, (5) Poor) [26]. The respondents also reported the expected delivery date and whether breastfeeding had been started after giving birth to the child. Pregnancy duration in days was calculated using parent-reported due date based on calculated date of conception (ie, 280 days of pregnancy) and date of birth of the child. In the control and intervention groups, the mother completed the questionnaire for both maternal and paternal characteristics in 93.14% (1030/1094) and 94.65% (954/1008) of cases, respectively (*P*=.34).

The eHealth module contained questions regarding usability of the module, including the ease of use and whether information regarding health-related behaviors was conveyed in a pleasant manner.

### Statistical Analyses

To compare the characteristics between the intervention group and the control group, we used either the Student’s t-test (for continuous variables) or the chi-square test (for categorical variables) [27].

Consistent with the data analyses described by Raat et al [8], the following primary outcomes were evaluated at follow-up (ie, when the child was 36 months of age): breakfast daily, activity and outdoor play, sweetened beverages, screen time (ie, TV watching and/or computer use), BMI, and overweight/obesity prevalence. Intention-to-treat analyses were performed using generalized linear mixed models or linear mixed models.

Here, we present the results of three regressions models. The first model did not include a correction for cluster (YHC team); the second model included corrections for cluster; and the third model included corrections for cluster and covariates [28,29]. Research condition (ie, intervention vs control) was entered in the models as an independent variable. Each primary outcome was evaluated as a dependent variable in a distinct model. With respect to the model evaluating BMI at follow-up, the outcome used was the value obtained when the child was as close to 36 months of age as possible (range: 33 to 40 months). Birth weight was added to the models for predicting BMI and BMI-SDS to take into account the impact of birth weight on BMI in later life [28,30]. Where available, each model was corrected for the previous assessment of the outcome at 14 months (for details, see Multimedia Appendix 3).

The following variables were evaluated as potential confounders: (1) pregnancy duration and birth weight; (2) maternal BMI; (3) maternal HRQoL and well-being (ie, self-rated health); and (4) maternal age, education level, and ethnic background [8]. A variable was considered a potential confounding factor when it was associated with the outcome and when it was distributed unequally between the intervention and control groups [27]. Both the child’s ethnic background and the mother’s country of birth fulfilled the criteria for a potential confounding factor. The child’s ethnic background was therefore added to the model as a covariate [27]. In addition, the models were corrected for the age of the child at the time of the follow-up assessment in order to correct for differences in the age at assessment [8,31,32].

Residuals followed a skewed distribution, and health-behavior assessments were log transformed for both the baseline and follow-up variables. Daily breakfast was dichotomized into daily breakfast yes/no because of non-normal distributed residuals after log transformation. Log transformation was performed using the natural logarithm. A constant of 0.01 was added because of zero values; activity n=1, sweet beverages n=192, and screen time n=25. The effect of research group can be interpreted by exponentiation of the coefficient as an approximate percent change in the outcome; percent change in the intervention group=100 x (exp[B]−1).

Subsequently, we evaluated moderation of the intervention effect by child’s gender and ethnic background, as well as the mother’s education level and weight status [8]. An interaction term between research condition and the potential moderating variable was added to the third multilevel regression model (ie, the model that was corrected for covariates) and was considered significant at *P*<.10 [32,33]. Stratified analyses were performed only when the interaction term reached this level of significance, and only statistically significant stratified analyses were presented. An overview of all interaction terms can be found in Multimedia Appendix 4.

In addition to the data analyses described in the study protocol [8], the following analyses were performed. Linear mixed models were used to evaluate the effect of the intervention on the longitudinal development of the child’s BMI from one month until 36 months of age. The model was adjusted for birth weight, the child’s age at each measurement, the child’s ethnic background, and an interaction term between age and birth weight. To evaluate whether BMI developed differently between the intervention and control groups, an interaction term between age and research condition was added to the models and evaluated at *P*<.10. To illustrate the development of BMI over time, Multimedia Appendix 5 presents the splined average BMI of the children versus age for both study groups.

To gain insight into the characteristics of the study participants who were lost to follow-up, we used descriptive statistics to compare the age, country of birth, living situation, and education levels of the mothers who remained in the study through the follow-up measurement (n=1543) with the mothers who were lost to follow-up (n=559). In addition, to get a deeper understanding of the characteristics of the mothers who completed the eHealth module, we also evaluated the abovementioned characteristics between the mothers who completed the eHealth module (n=651) and the mothers who did not complete the eHealth module (n=357) when the child was 18 months of age.

Descriptive statistics were analyzed using the Statistical Package for the Social Sciences (SPSS) version 21.0 (IBM Corp). Generalized linear mixed models and other linear mixed models were performed using Statistical Analysis Software (SAS) version 9.4 (SAS Institute Inc).

## Results

### Study Population

Table 1 summarizes the characteristics of the study population. Compared with the children in the control group, the children in the intervention group were slightly but significantly older at the time of inclusion. Moreover, the intervention group contained a significantly higher percentage of children of Dutch ethnic background and a significantly higher percentage of mothers who were born in the Netherlands.

**Table 1:** Characteristics of the study population at study inclusion (n=2102 parents).

Characteristics			Total study population (n=2102)	Control group (n=1094)	Intervention group (n=1008)	*P*^a^
**Child characteristics**						
	**Sex (missing n=31), n (%)**					
		Male	1048 (50.60)	567 (52.55)	481 (48.49)	.07
	Age at inclusion in months (missing n=15), mean (SD^b^)		0.51 (0.88)	0.47 (0.80)	0.55 (0.95)	.03
	Age at the follow-up assessment in months (missing n=810), mean (SD)		36.84 (2.37)	36.87 (2.47)	36.81 (2.27)	.67
	Birth weight in kilograms^c^ (missing n=14), mean (SD)		3458.41 (525.73)	3453.60 (528.41)	3463.61 (523.03)	.66
	**Ethnic background^d^ (missing n=4), n (%)**					
		Dutch	1713 (81.65)	862 (78.94)	851 (84.59)	.001
	**Family situation (missing n=30), n (%)**					
		Both parents	2029 (97.92)	1053 (97.68)	976 (98.19)	.44
**Maternal characteristics**						
	Age in years (missing n=28), mean (SD)		30.88 (4.33)	30.99 (4.41)	30.75 (4.24)	.21
	Pregnancy duration in days (missing n=66), mean (SD)		277.38 (10.72)	277.01 (10.96)	277.77 (10.44)	.11
	**Started breastfeeding? (missing n=34), n (%)**					
		Yes	1584 (76.60)	795 (73.75)	789 (79.70)	.002
	BMI^e^ in kg/m^2^ (missing n=152), mean (SD)		25.09 (4.21)	25.26 (4.45)	24.91 (3.88)	.06
	**Country of birth (missing n=7), n (%)**					
		The Netherlands	1931 (92.17)	984 (90.36)	947 (94.14)	.001
	**Education level (missing n=29), n (%)**					.26
		Low	272 (13.12)	148 (13.77)	124 (12.43)	.15
		Mid	725 (34.97)	359 (33.40)	366 (36.67)	.80
		High	1076 (51.91)	568 (52.84)	508 (50.90)	.07
	**Employment status (missing n=67), n (%)**					
		Employed	1683 (82.70)	867 (82.3)	816 (83.18)	.60
	**Self-rated health^f^ (missing n=713), n (%)**					
		Very good or excellent	790 (56.88)	395 (57.92)	395 (55.87)	.45

^a^The *P*-value is based on an independent Student’s t-test (for continuous variables) or the chi-square test (for categorical variables) to analyze the difference between the control and intervention groups.

^b^SD: standard deviation.

^c^Birth weight was collected by the Youth Health Care professional; if missing, this value was obtained from the parent’s inclusion questionnaire.

^d^Ethnic background of the child was based on the grandparents’ country of birth as described by Statistics Netherlands. If one or both grandparent were born outside the Netherlands, the parents were categorized as non-Dutch. If one or both of the parents were categorized as non-Dutch, the child was also categorized as being of non-Dutch origin.

^e^BMI: body mass index.

^f^Self-rated health of the parent when the child was 36 months of age.

**Table 2:** Descriptive summary of the primary outcomes measured at 14 months and at follow-up.

Primary outcomes	14 months^a^			36 months^b^		
	Control group	Intervention group	*P*^c^		Control group	Intervention group	*P*^c^
Daily breakfast 7 days/week, %	98.0	98.0	.55		96.7	98.3	.03
Activity, hours/day, mean (SD^d^)	1.91 (1.30)	1.88 (1.24)	.72		2.56 (1.40)	2.68 (1.13)	.19
Sweetened beverages, glasses/day, mean (SD)	1.34 (1.16)	1.39 (1.24)	.39		2.31 (1.51)	2.10 (1.28)	.003
Screen time^e^, hours/day, mean (SD)					1.22 (0.92)	1.05 (0.74)	<.001
BMI^f^, mean (SD)	16.75 (1.25)	16.83 (1.24)	.18		15.66 (1.29)	15.78 (1.23)	.12
BMI-SDS^g^, mean (SD)	-0.25 (0.96)	-0.17 (0.94)	.10		-0.17 (1.02)	-0.06 (1.01)	.048
Overweight or obesity^h^, %					3.99	4.77	.51

^a^Number of missing values range 448 to 541

^b^Number of missing values range 559 to 915

^c^*P* value from independent t-test for continuous variables and chi-square tests for categorical variables.

^d^SD: standard deviation.

^e^Not assessed before 36 months.

^f^BMI: body mass index.

^g^BMI-SDS: body mass index-standard deviation score.

^h^Percentage of overweight and obesity defined by the international age-and gender specific cutoff values; cannot be defined before the age of 24 months.

### Primary Outcome Measures

Table 2 summarizes the descriptive results and statistical analyses of the health-related behaviors, BMI, and the prevalence of overweight/obesity among the children in the intervention and control group at both 14 and 36 months of age.

At 36 months of age, significantly more children in the intervention group ate breakfast daily (*P*=.03) as compared with the control group. In addition, children in the intervention group drank less sweetened beverages (*P*=.003). Moreover, children in the intervention group had less screen time compared with the children in the control group (*P*<.001).

At 36 months of age, the BMI of children in the control group and the intervention group was 15.66 (SD 1.29) and 15.78 (SD 1.23), respectively (*P*=.12). Children in the intervention group had a BMI-SDS closer to the reference population as compared with the children in the control group (−0.06, SD 1.01 vs −0.17, SD 1.02, between group difference *P*=.048). The percentage of children classified as being overweight or obese was similar between the two study groups (*P*=.51).

The results of the regression analyses are summarized in Table 3, in which we evaluated the effect of the E-health4Uth Healthy Toddler intervention compared with the control group at 36 months of age. Without correction for cluster or covariates, children in the intervention group decreased 13.06% in screen time at follow-up compared with the control group (95% CI −20.55 to −3.92, *P*=.005); after correction for cluster and covariates the coefficient was no longer significant. No significant effects were found with respect to the other health-related behaviors.

With regard to BMI the third model (ie, the model corrected for potential covariates) revealed a beta value for BMI at follow-up of .10 (95% CI −0.15 to 0.36) for the children in the intervention group as compared with the children in the control group; for BMI-SDS, beta was .12 (95% CI −0.091 to 0.33). With respect to overweight/obesity, the odds ratio at 36 months for the children in the intervention group was 0.79 (95% CI 0.44-1.43) compared with the children in the control group.

### Longitudinal Development of BMI

In addition, we analyzed BMI and BMI-SDS longitudinally. The interaction term between the study group and age was not significant for either BMI or BMI-SDS (*P*=.27 and *P*=.39, respectively), indicating that the relationship between BMI and age did not differ significantly between the intervention and control group. Multimedia Appendix 5 shows the splined average BMI values of the children in the intervention and control groups.

**Table 3:** Results of the three models evaluating primary outcomes among the children at 36 months of age.

Primary outcomes at 36 months	Model 1^a,b^		Model 2^c,b^		Model 3^d,b^	
	Intervention group^e^	*P*	Intervention group^e^	*P*	Intervention group^e^	*P*
Daily breakfast (yes), OR^e^ (95% CI)^f^	1.55 (0.74 to 3.25)	.25	1.55 (0.72 to 3.34)	.25	1.31 (0.56 to 3.10)	.52
Activity and outdoor play^g^, hours/day, beta (95% CI)	.04 (−0.02 to 0.10)	.18	.04 (−0.05 to 0.13)	.38	.05 (−0.04 to 0.15)	.29
Sweetened beverages^g^, glasses/day, beta (95% CI)	−.14 (−0.31 to 0.03)	.11	−.14 (−0.31 to 0.03)	.11	−.16 (−0.34 to 0.03	.10
Screen time^g^, hours/day, beta (95% CI)	−.14 (−0.23 to −0.04)	.005	−.09 (−0.27 to 0.08)	.30	−.07 (−0.25 to 0.12)	.47
BMI^h^, beta (95% CI)	.11 (−0.03 to 0.25)	.13	.11 (−0.15 to 0.36)	.40	.10 (−0.15 to 0.36)	.43
BMI-SDS^i^, beta (95% CI)	.11 (0.00 to 0.22)	.06	.12 (−0.09 to 0.33)	.26	.12 (−0.09 to 0.33)	.28
Overweight or obesity^j^, OR (95% CI)	0.83 (0.48 to 1.46)	.52	0.83 (0.46 to 1.49)	.52	0.79 (0.44 to 1.43)	.43

^a^Model 1: corrected for the previous assessment of the outcome (where available).

^b^Models evaluating BMI, BMI-SDS and % overweight or obesity are corrected for birth weight of the child.

^c^Model 2: corrected for cluster Youth Health Care (YHC) team and the previous assessment of the outcome (where available).

^d^Model 3: corrected for cluster (YHC team), the previous assessment of the outcome (where available), the child’s ethnic background, and the child’s precise age at follow-up.

^e^OR: odds ratio.

^f^The estimated coefficients and their 95% confidence interval (95% CI) are given for the children in the intervention group relative to the children in the control group.

^g^The previous assessment of the outcome (where available) and the outcome at follow-up were log transformed

^h^BMI: body mass index.

^i^BMI-SDS: body mass index-standard deviation score.

^j^Percent overweight or obese is based on the definition reported by Cole et al [7].

### Evaluation of Moderating Factors

After observing a significant interaction term between potential moderators and the study groups, we performed stratified analyses. Our analysis revealed that the boys in the intervention group were more likely to eat breakfast daily compared with the boys in the control group at follow-up (OR 10.20; 95% CI 1.75-88.60). Non-Dutch children in the intervention group were 25.86% more active at follow-up compared with the non-Dutch children in the control group (95% CI 0.80-56.83, *P*=.04). Children with low educated mothers in the intervention group decreased 46.74% in screen time as compared with children of low educated mothers in the control group (95% CI −70.48 to −4.88, *P*=.03). Children of mothers with a BMI categorized as “normal” in the intervention group drank 36.24% less sweet beverages at follow-up compared with the children of “normal” weight mothers in the control group (95% CI −54.16 to −10.42, *P*=.009). Children of mothers with a BMI classified as overweight or obese in the intervention group showed 26.11% less screen time at follow-up compared with children of mothers with a similar BMI categorization in the control group (95% CI −46.74 to −2.96, *P*=.03).

### Other Outcome Measures

#### Parents’ Evaluation of the Intervention

The eHealth module was completed primarily by the mother when the children were 18 and 24 months of age (626/651, or 96.2%, and 610/638 missing n=2, or 95.9%, respectively). The parents also reported that they found the eHealth module easy to use (470/651 missing n=81, or 82.5%, and 469/638 missing n=67, or 82.1%, when the children were 18 and 24 months of age, respectively). At 18 months, 60.1% (342/651 missing n=82) and 61.6% (350/651 missing n=83) of parents appreciated receiving information regarding physical activity and nutrition, respectively, via the eHealth module.

#### Evaluation of the Characteristics of Parents Who Were Not Lost to Follow-Up and Parents Who Completed the eHealth Module

On an average, the mothers who participated through to the follow-up time point were older than the mothers who were lost to follow-up (31.11, SD 4.18 vs 30.24, SD 4.65 years, respectively; *P*<.001). In addition, a higher percentage of these mothers were born in the Netherlands (93.05%, 1433/1543 vs 89.73%, 498/559, respectively; *P*=.009) and lived with their partner (98.55%, 1499/1543 vs 96.19%, 530/559, respectively; *P*=.001). Finally, a higher percentage of these mothers had a higher level of education (56.04%, 858/1543 vs 40.22%, 218/559, respectively; *P*<.001).

In total, 651 out of 1008 (64.58%) parents completed the eHealth module when their child was 18 months of age, compared with 357 out of 1008 (35.42%) parents who did not complete the eHealth module at this time point. On an average, the mothers who completed the eHealth module at this time point were older than the mothers who did not complete the module (31.06, SD 4.06 vs 30.19, SD 4.50 years, respectively; *P*=.003). In addition, a higher percentage of these mothers were born in the Netherlands (95.4%, 621/651 vs 91.8%, 326/355, respectively; *P*=.17) and had a high level of education (58.7%, 380/647 vs 36.5%, 128/351, respectively; *P*<.001).

## Discussion

### Principal Findings

In this study, we evaluated the effects of the E-health4Uth Healthy Toddler intervention on the child’s health-related behaviors and BMI [8]. The E-health4Uth Healthy Toddler intervention provided parents with personalized advice regarding overweight-related health behaviors for their child at the ages of 18 and 24 months. This advice was then discussed between the parent and YHC professional during a regular face-to-face well-child visit. Our analysis revealed no significant effect of the intervention on health-related behaviors, BMI, BMI-SDS, or the prevalence of overweight/obesity. Significant interaction terms indicated that the intervention had positive effects on subgroups of children, namely boys, non-Dutch children, children of low educated mothers, and children from mothers with a “normal” and overweight and obese classified BMI. The intervention was generally appreciated by the parents.

### Interpretation

This study adds to the overall knowledge base regarding educating parents in order to optimize the healthy behaviors of young children [34]. This is one of the first studies to combine an eHealth intervention with personalized counseling at a YHC setting, particularly with respect to both diet and physical activity. To date, relatively few interventions have been performed among children 2 years of age or younger, and the primary focus of these studies was parental feeding practices such as breastfeeding or the management of specific problems [34,35]. Similar interventions performed in primary care settings and/or well-child clinics have been evaluated among parents of slightly older children (5-10 years of age) [21,36,37]; these studies reported small beneficial effects of the intervention on the child’s health-related behaviors and BMI. Thus, in theory the relatively younger children in our study may require a longer follow-up period in order to observe significant effects, particularly given that the E-health4Uth Healthy Toddler intervention focuses on primary prevention and therefore, the promotion of healthy behaviors and BMI. Interventions specifically designed for young children who are at a risk for becoming overweight—or are already overweight or obese—may benefit from such an intervention at an earlier age.

Our results show that at baseline and at 36 months of age, the children in the intervention group had a higher BMI as compared with the children in the control group; we currently have no explanation for this finding. In fact, compared with the age- and gender-matched reference population measured in 1980, the children in the entire study sample had relatively healthier BMI-SDS values. It is important to note that the Dutch reference population values from 1980 were measured just before the overweight epidemic [1]. On an average, the age-and gender-corrected standardized BMI values of the children in our study were only slightly higher than the reference values. Interestingly, the children in our sample were generally taller than the children in the 1980 reference population; however, van Dommelen et al [38] reported that taller children tend to be overweight more often than shorter children. Another explanation might be the fact that our sample contained a relatively higher number of mothers with high education; such parents tend to have less overweight children [39]. The finding of this relatively healthy sample, however, would not necessarily influence the results obtained between the two groups, although it could have limited the potential effects that could have been observed. Nevertheless, the need for an intervention that is independent of the child’s weight status is supported by the prevalence of unhealthy lifestyle behaviors among the children in our sample.

The importance of factors in early life that determine the development of overweight among children reflects the need to further develop and optimize interventions designed specifically for parents of young children [34,39,40]. In this respect, some of the elements in the E-health4Uth Healthy Toddler intervention may be helpful. In order to be feasible for use in a wide range of YHC settings, and to minimize the burden on the parents, a relatively low-intensity intervention was designed; this intervention includes two eHealth modules, with personalized advice for parents and a discussion of this advice during the two regular well-child visits at a YHC organization. This approach has two advantages. Firstly, the combination of Web-based personalized advice together with a face-to-face well-child visit at the YHC organization might increase the intervention’s effectiveness [9-11,41]. Importantly, the E-health4Uth intervention provides the parents with information regarding their child’s health-related behaviors before the well-child visit. Thus, the parents can read the information in advance at their leisure, which may increase the likelihood that the information will be well-received during the well-child visit [42]. Secondly, if the YHC professional utilizes the advice that the parents received before the well-child visit, the visit can be designed more efficiently to discuss the child’s most relevant health-related behaviors. The personalized advice is based on the parents’ knowledge, and the child’s health-related behavior. Because the YHC professional receives a copy of the advice given to the parents, this information can be used to customize the information provided to the parents during the well-child visit [42,43]. During the well-child visit, motivational interviewing techniques are used by professionals to help the parents effect change in the child’s behavior; the use of these techniques may contribute to the effectiveness of interventions [44,45]. Finally, the intervention focuses on the child’s health-related behaviors rather than the child’s weight status. Interventions performed in primary care (or comparable) settings often encounter difficulty with respect to motivating the parents to change their health-related behaviors, usually due to the fact that many parents either underestimate or overestimate their child’s weight status [42,43,46,47]. Moreover, many parents cannot remember the information and/or advice that was provided after their child’s height and weight were measured [42]. Therefore, the focus on the child’s health-related behaviors in the E-health4Uth Healthy Toddler intervention is another possible advantage and may create a reason to discuss the child’s overweight status, if needed.

Other elements of the E-health4Uth Healthy Toddler intervention can be optimized and/or revised. For example, to increase its effectiveness, the intervention can be revised to give specific advice to parents of children beyond 24 months and/or to at an earlier age (ie, before 18 months) [48,49]. Providing parents with information regarding healthy lifestyle behaviors can have long-term benefits with respect to the targeted health-related behavior, as shown previously by Talvia et al [50]. Such a continuum of interventions focusing on parents from the birth of their child through childhood seems promising and is well-suited to the early life systems approach, which includes interventions during pregnancy, infancy, and the toddler years [40]. In this type of approach, one of the key pathways includes the behavior, policy, and practice of health care providers to promote a healthy lifestyle [40,51] by targeting several risk factors for overweight (eg, maternal smoking, overweight, and sleeping patterns) [39,52]. In this respect, the E-health4Uth Healthy Toddler intervention meets the criteria for an early life systems approach.

The E-health4Uth Healthy Toddler intervention offers the opportunity to provide parents with important messages that are strengthened by personal counseling with the YHC professional [10]. Such messages can be delivered to parents more effectively when professionals use motivational interviewing techniques [45]. However, to ensure the adequate and proper use of these techniques, continuous practice by the professional and repeated training sessions can help improve future interventions [45,53]. Also, the integrity of the use of motivational interviewing techniques may be evaluated more closely using specific instruments such as the Motivational Interviewing Treatment Integrity Code [54].

Future studies should be designed to test potential beneficial effects of combining an eHealth module with face-to-face counseling among various subgroups such as children of less-educated parents, children of non-Dutch ethnic background, and gender subgroups. Therefore, the E-health4Uth Healthy Toddler intervention can be easily adapted for use in these subgroups [46,55]. Moreover, the intervention can be revised in order to improve other characteristics of the target population. For example, advice regarding physical activity can be optimized by providing parents with local outdoor play opportunities for their children based on their zip code and/or geographic region. This type of specific, personalized advice could be added to the eHealth module; alternatively, it could be provided during the counseling session with the YHC organizations themselves, as these organizations operate within local communities and are keenly aware of local activities such as after-school physical activity programs.

### Strengths and Limitations

The strength of our approach was our collaboration with the Dutch YHC organizations, which enabled us to perform a large-scale cluster RCT using Web-based eHealth combined with face-to-face counseling by community-based pediatric health care professionals engaged in daily practice. This approach also provided the opportunity to obtain a large dataset of objectively measured height and weight outcomes for the children whose parents participated in the study. In addition, the response rate among the parents was relatively high (approximately 75%), enabling us to conduct a relatively thorough evaluation of the intervention’s effects on health-related behaviors and BMI.

A limitation of the study was the transformation of outcome variables, namely activity, sweetened beverages, and screen time. We performed sensitivity analyses using the dichotomized version of these primary outcome variables (data not shown) and similar results, that is, no significant differences between intervention and control group, were observed. Another possible limitation of this study was the use of self-report questionnaires, in which parents may have underestimated or overestimated their child’s behavior. However, given that the same assessment materials were used in both the intervention and control groups, this effect—if present—would not likely have affected our results. Moreover, parents with a higher education were more likely to participate in the follow-up measure and in the intervention. Even though the sample may not perfectly represent the general population in the Netherlands, the sample size was sufficiently large for us to evaluate the potential moderating effects among higher and lower educated parents. Regardless, future research and implementation of these types of interventions should emphasize on hard-to-reach lower educated parents. One option for reaching these parents is to provide the eHealth module in the waiting room when the parents arrive for their well-child visit; this approach would also create an opportunity for parents to ask questions to the nurse and/or physician directly after completing the eHealth module.

### Conclusions

This large cluster RCT evaluated the E-health4Uth Healthy Toddler intervention, which combines an eHealth module with face-to-face interaction between parents and YHC professionals. Our analysis revealed limited evidence with regard to health behavior and overweight prevention in young children. However, some indications for effects among subgroups of parents and children, such as less-educated parents, were observed. The E-health4Uth Healthy Toddler intervention is relatively easy to implement in community medicine and preventive pediatric practice and can serve as an important addition to current medical guidance and health-promoting practices. This low-intensity intervention can be added to regular care and may save health care professionals valuable time that can be used to focus on health-related behaviors that are determined to be most relevant by the eHealth module. The combination of personalized advice and face-to-face counseling likely increases the effectiveness of this type of intervention [11]. The results of our analysis suggest that some elements of the E-health4Uth Healthy Toddler intervention can be improved further. For example, the intervention can be adapted for parents of children younger than 18 months and children older than 24 months [40]. Thus, information regarding the child’s healthy lifestyle behaviors will be conveyed to parents repeatedly, potentially improving its long-term benefits [48]. The intervention can be offered in a pediatric setting, as well as in other settings such as general practice. Importantly, the advice provided to the parents should be specific to the age of the child. In summary, this eHealth module can be adapted relatively easily to reach out to a wide range of parents, thereby contributing to reducing the prevalence of childhood overweight and obesity.

## Appendix

Multimedia Appendix 1 E-health4Uth Healthy Toddler intervention example advise sweetened beverages.

Multimedia Appendix 2 E-health4Uth Healthy Toddler intervention example advise activity.

Multimedia Appendix 3 Summary of the items for assessing the children’s health-related behaviors.

Multimedia Appendix 4 Overview of interaction terms.

Multimedia Appendix 5 Splined average of the BMI values in the intervention and control group.

Multimedia Appendix 6 CONSORT E-HEALTH checklist (V1.6.1).

## Abbreviations

BMI: body mass index
BMI-SDS: body mass index-standard deviation score
HRQoL: health-related quality of life
OR: odds ratio
RCT: randomized controlled trial
SAS: Statistical Analysis Software
SD: standard deviation
SPSS: Statistical Package for the Social Sciences
TV: television
YHC: youth health care
