# Supplementary figures and images for: Personalized Web-Based Advice in Combination With Well-Child Visits to Prevent Overweight in Young Children: Cluster Randomized Controlled Trial
Source: J Med Internet Res. 2017 Jul 27;19(7):e268. doi: 10.2196/jmir.7115 (PMC5553002; doi:10.2196/jmir.7115)

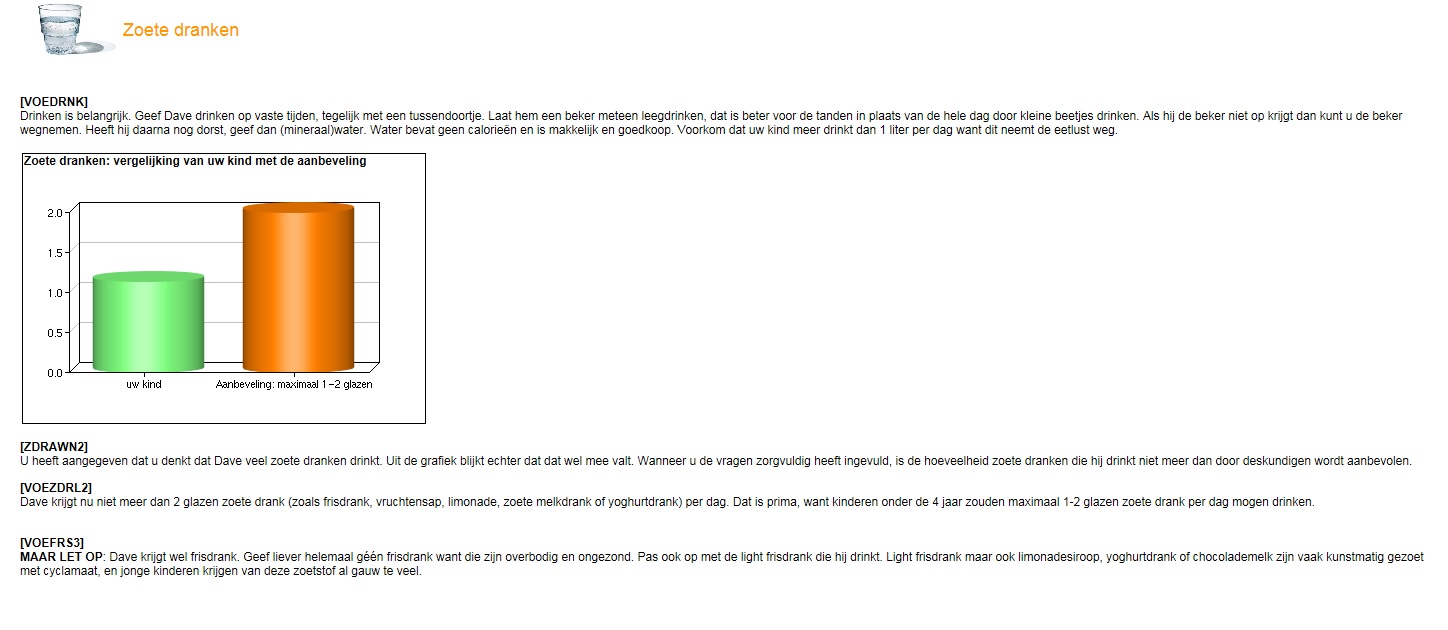

Supplement: Multimedia Appendix 1 [file jmir_v19i7e268_app1.jpg]

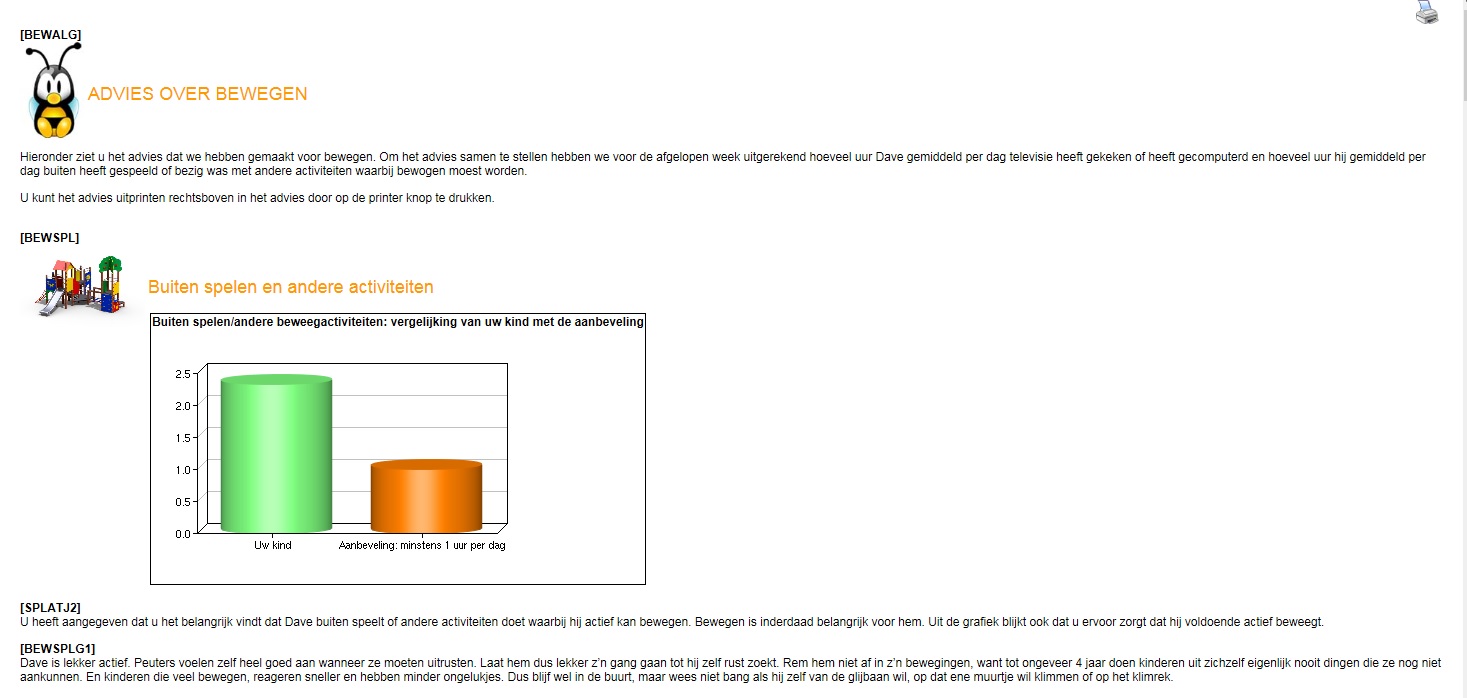

Supplement: Multimedia Appendix 2 [file jmir_v19i7e268_app2.jpg]

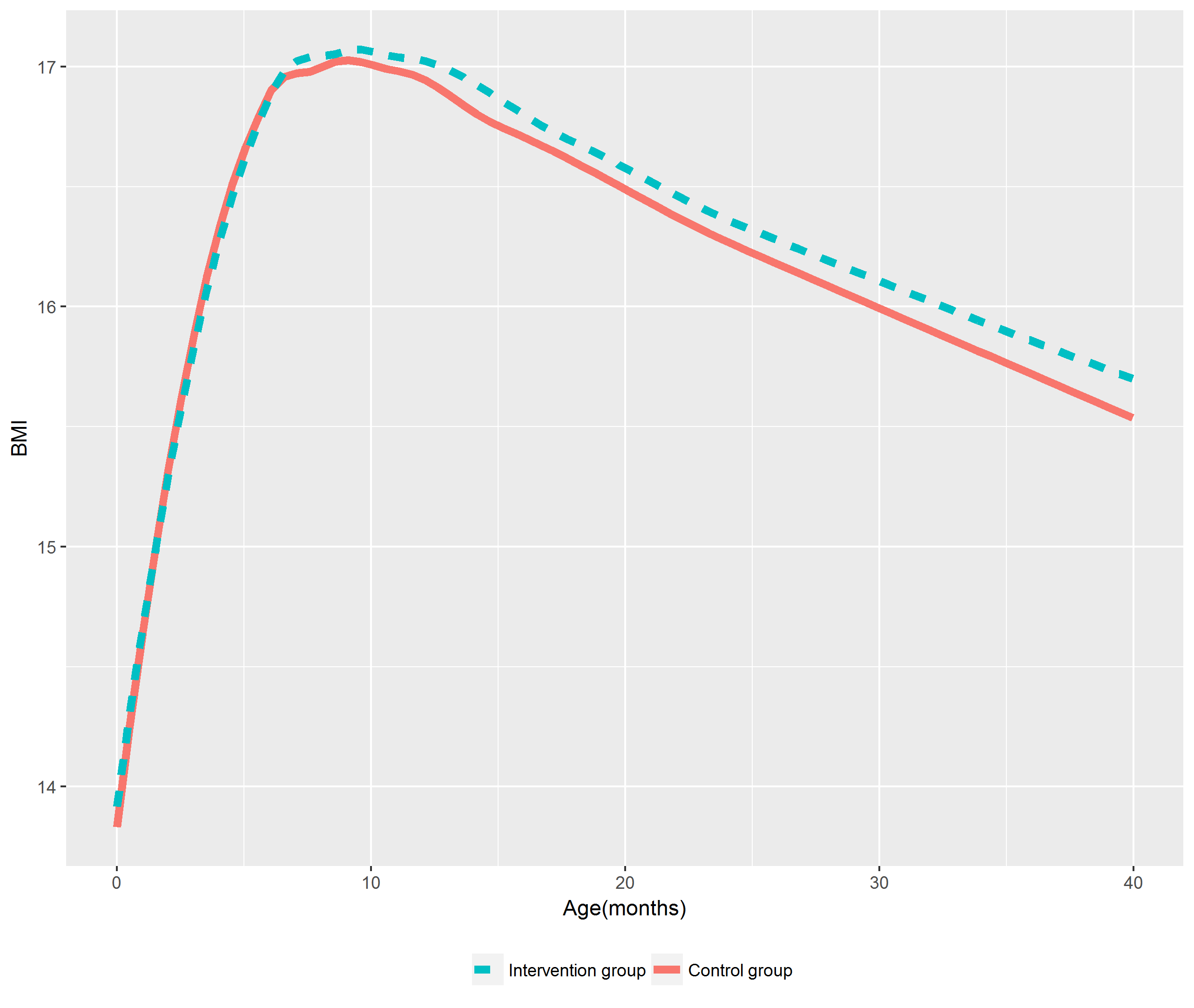

Supplement: Multimedia Appendix 5 [file jmir_v19i7e268_app5.png]
