# Supplementary material for: Personalized Web-Based Advice in Combination With Well-Child Visits to Prevent Overweight in Young Children: Cluster Randomized Controlled Trial
Source: J Med Internet Res. 2017 Jul 27;19(7):e268. doi: 10.2196/jmir.7115 (PMC5553002; doi:10.2196/jmir.7115)
Supplement: Multimedia Appendix 3 [file jmir_v19i7e268_app3.pdf]

Multimedia appendix 3. Summary of the items for assessing the children's health-related behaviors.

Health behavior outcome	Questionnaire (age of the child in months)	Item	Response scale
Daily activity and outdoor play			
Daily physically days/week)	14, 36	How many days a week does your child perform active activities (e.g. swimming, move on music, walking to the shop). Regular play in the home does not count.	(almost) never, 1 day a week, 2 days a week, 3 days a week, 4 days a week, 5 days a week, 6 days a week, every day
Physically active hours /day	14, 36	How much time a day is your child active with these activities?	Less than 30 minutes, 30 minutes to 1 hour, 1 to 2 hours, 2 to 3 hours, more than 3 hours, not applicable
Daily outside play days/week	14, 36	How many days a week does your child go outside to play?	(almost) never, 1 day a week, 2 days a week, 3 days a week, 4 days a week, 5 days a week, 6 days a week, every day
Outside play hour/day	14, 36	How much time does your child spent playing outside a day?	Less than 30 minutes, 30 minutes to 1 hour, 1 to 2 hours, 2 to 3 hours, more than 3 hours, not applicable
Breakfast			
Daily breakfast days/week	14, 36	How many days of the week does your child have breakfast?	Number of days: 0 to 7
Sweetened beverages (e.g. carbonated soft drinks, non-carbonated soft drinks, fruit juices, sport- and energy drinks, milk and yoghurt drinks, tea or water with sugar)			
Sweetened beverages glasses/ weekday	14, 36	How many glasses of sweetened beverages does your child consume on	Less than 1, 1, 2, 3, 4, 5, 6, 7, 8, 9 or more

		average on a week day ?	
Sweetened beverages glasses/ weekend day	14, 36	How many glasses of sweetened beverages does your child consume on average on a weekend day?	Less than 1, 1, 2, 3, 4, 5, 6, 7, 8, 9 or more
Screen time (TV viewing and computer use)			
TV viewing hours/ weekday	36	How much time per day does your child on average watch television on a week day?	Less than 30 minutes, 30 minutes to 1 hour, 1 to 2 hours, 2 to 3 hours, 3 to 4 hours, 4 to 5 hours, 5 to 6 hours, more than 6 hours, not applicable
TV viewing hours/ weekend day	36	How much time per day does your child on average watch television on a weekend day?	Less than 30 minutes, 30 minutes to 1 hour, 1 to 2 hours, 2 to 3 hours, 3 to 4 hours, 4 to 5 hours, 5 to 6 hours, more than 6 hours, not applicable
Computer use hours/ weekday	36	How much time per day does your child on average spent playing computer games on a week day?	Less than 30 minutes, 30 minutes to 1 hour, 1 to 2 hours, 2 to 3 hours, 3 to 4 hours, 4 to 5 hours, 5 to 6 hours, more than 6 hours, not applicable
Computer use hours/ weekend day	36	How much time per day does your child on average spent playing computer games on a weekend day?	Less than 30 minutes, 30 minutes to 1 hour, 1 to 2 hours, 2 to 3 hours, 3 to 4 hours, 4 to 5 hours, 5 to 6 hours, more than 6 hours, not applicable
