# Supplementary material for: Personalized Web-Based Advice in Combination With Well-Child Visits to Prevent Overweight in Young Children: Cluster Randomized Controlled Trial
Source: J Med Internet Res. 2017 Jul 27;19(7):e268. doi: 10.2196/jmir.7115 (PMC5553002; doi:10.2196/jmir.7115)
Supplement: Multimedia Appendix 4 [file jmir_v19i7e268_app4.pdf]

Multimedia appendix 4. Overview of interaction terms.

	Gender child	Ethnic background child	Maternal education level	Maternal overweight
	P [a]	*P*[a]	*P*[a]	*P*[a]
Daily breakfast (yes/no)	.01	.71	not possible	.71
Activity and outside play, hours/day[b]	.93	.02	.55	.93
Sweetened beverages, glasses/day[b]	.25	.59	.72	.08
Screen time, hours/day[b]	.29	.96	<.001	.099
BMI	.95	.67	.29	.67
BMI-SDS	.92	.65	.35	.70
% Overweight or obesity	.77	.95	.15	.72

[a] *P*-value of the interaction term potential moderator * research condition, derived from the full model corrected for potential confounders.

[b] Log transformed.
